# Radiation-induced formation of purine lesions in single and double stranded DNA: revised quantification

**DOI:** 10.3389/fchem.2015.00018

**Published:** 2015-03-20

**Authors:** Michael A. Terzidis, Carla Ferreri, Chryssostomos Chatgilialoglu

**Affiliations:** ^1^Istituto per la Sintesi Organica e la Fotoreattività, Consiglio Nazionale delle RicercheBologna, Italy; ^2^Institute of Nanoscience and Nanotechnology, National Centre for Scientific Research “Demokritos”Athens, Greece

**Keywords:** gamma radiation, free radicals, DNA damage, cyclopurines, 8-oxo-dG, LC-MS/MS

## Abstract

The formation of oxidative lesions arising from double stranded DNA damage is of major significance to chemical biology from the perspective of application to human health. The quantification of purine lesions arising from γ-radiation-induced hydroxyl radicals (HO^•^) has been the subject of numerous studies, with discrepancies on the measured 5′,8-cyclo-2′-deoxyadenosine (cdA) and 5′,8-cyclo-2′-deoxyguanosine (cdG) lesions reported by different groups. Here we applied an ameliorative protocol for the analysis of DNA damage with quantitative determination of these lesions via isotope dilution liquid chromatography coupled with tandem mass spectrometry. Tandem-type purine lesions were quantified along with 7,8-dihydro-8-oxo-2′-deoxyguanosine (8-oxo-dG) and 7,8-dihydro-8-oxo-2′-deoxyadenosine (8-oxo-dA) in single and double stranded DNA, generated during DNA exposure to diffusible HO^•^ radicals in the absence or presence of physiological levels of oxygen. The cdA and cdG lesions in absence of oxygen were found ~2 times higher in single than double stranded DNA, with 5′*R* being ~6.5 and ~1.5 times more predominant than 5′*S* in cdG and cdA, respectively. Interestingly, in the presence of 5% molecular oxygen the R/S ratios are retained with substantially decreased yields for cdA and cdG, whereas 8-oxo-dA and 8-oxo-dG remain nearly constant. The overall lesion formation follows the order: 8-oxo-dG >> 8-oxo-dA > 5′*R*-*cdG* > 5′*R*-*cdA* > 5′*S*-*cdA* > 5′*S*-cdG. By this method, there was a conclusive evaluation of radiation-induced DNA purine lesions.

## Introduction

**Figure 1:**
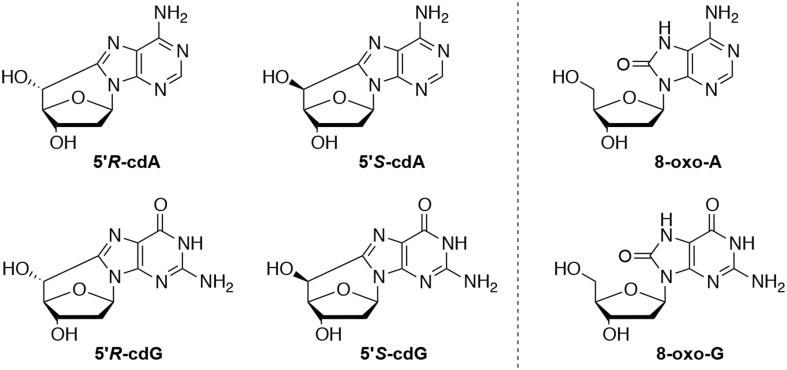
**Left:** 5′,8-Cyclo-2′-deoxyadenosine (cdA) and 5′,8-cyclo-2′-deoxyguanosine (cdG) lesions; **Right:** 7,8-dihydro-8-oxo-2′-deoxyguanosine (8-oxo-dG) and 7,8-dihydro-8-oxo-2′-deoxyadenosine (8-oxo-dA) lesions.

Genetic information of all living organisms is stored in DNA, a polymer consisting of 2′-deoxynucleosides. Reactive species, resulting from metabolic reactions, stresses, environmental conditions, presence of chemicals, etc., can alter the integrity of this biopolymer. Among highly reactive species, the hydroxyl radicals (HO^•^) can lead to nucleobase modifications and to strand breaks. The H5′ of sugars in DNA have been found quite vulnerable with a 55% probability of abstraction by HO^•^, in respect to the rest of the sugar hydrogen atoms (Aydogan et al., 2002). This fact leads to the generation of peculiar lesions containing a carbon-carbon bond between the sugar and the purine, rather than to abasic sites (Chatgilialoglu et al., 2011a). 5′,8-Cyclo-2′-deoxyadenosine (cdA) and 5′,8-cyclo-2′-deoxyguanosine (cdG), in their 5′*S* and 5′*R* diastereomeric forms (Figure 1), have been identified among other DNA lesions in enzymatically digested cellular DNA of mammals (Chatgilialoglu et al., 2011a). Moreover it has been recently reported that aging causes accumulation of the cyclopurines in tissues, aging liver holding the first place in aging accumulated cyclopurines, followed by the kidney, while lower levels were found in the brain (Wang et al., 2011, 2012). It is worth noting that the cyclopurine lesions have been used in a recent study as reliable oxidative stress biomarkers in animal models focusing on the liver injury pathophysiology in Wilson's disease and pigmentation (Mitra et al., 2012; You et al., 2012). As matter of fact, oxidative artifacts during work-up of biological DNA samples are reported in the evaluation of 8-oxo-dG lesions, whereas cyclopurines are “pure” radical-derived products, and cannot derive from accidental oxidation of the material.

The repair efficiency of cyclopurine lesions has recently been studied in some aspects and more information is expected in the near future. These lesions are repaired by Nucleotide Excision Repair (NER) and not by Base Excision Repair (BER). HeLa cell extracts have been used in identical DNA sequences containing the cdA and cdG lesions giving a direct comparison of the relative NER efficiencies that was missing (Kropachev et al., 2014). Interestingly, the study revealed a higher repair efficiency, by a factor of ~2, for the 5′*R* over the 5′*S* cyclopurines, however the excision efficiency of the cdA and cdG lesions was found similar. In the same vein, extensive *in silico* analysis confirmed the correlation of the stacking impairment of the stereoisomer with their relative NER excision efficiency. Evidence was recently provided that both 5′*R*-cdA and 5′*S*-cdA in a CAG repeat tract causes CTG repeat deletion exclusively during DNA lagging strand maturation and BER activity (Xu et al., 2014). The latter study indicates that accumulation of cyclopurine lesions in the human genome can lead to trinucleotide repeat instability via a unique lesion bypass by pol β.

Increased levels of cdA and cdG are linked to NER mechanism deficiency and mutagenesis. Particularly, 5′*S*-cdG results in strong inhibition and block of the transcription in both *in vitro* and in mammalian cells as found by a competitive transcription and adduct bypass assay by a recent study (You et al., 2012).

Attempts to accurately determine the level of these lesions in DNA by enzymatic digestion followed by chromatographic methods coupled to mass spectrometric (MS) analyses are numerous. Results reported for irradiated samples of calf thymus DNA have been critically reviewed by us in 2011, underlining the need of further research for the potential involvement of these lesions in human health (Chatgilialoglu et al., 2011a). A detailed protocol revision for the quantification of purine lesions in DNA has been recently provided (Terzidis and Chatgilialoglu, in preparation). The objectives of this work were to apply our protocol based on the stable isotope-dilution tandem mass spectrometry technique using triple quadrupole for the quantification of HO^•^ radical induced cyclopurine and 8-oxopurine lesions within single- and double-stranded DNA in gamma-irradiated aqueous solutions.

## Materials and methods

### Chemicals

All the salts and solvents, activated calf thymus DNA, nuclease P1 from Penicillium citrinum, phosphodiesterase II, phosphodiesterase I from Crotalus Adamanteus venom, DNAse I, DNAse II, alkaline phosphatase from bovine intestinal mucosa, erythro-9-(2-hydroxy-3-nonyl)adenine hydrochoride (EHNA), benzonase 99%, deferoxamine mesylate salt, BHT and pentostatin, were obtained from Sigma (Taufkirchen, Germany and Milan, Italy) while the 3 and 100 kDa cut-off filters were purchased from Millipore (Bedford, USA). Distilled and deionized water (ddH_2_O) was purified by a Milli-Q system (Millipore, Bedford, USA). Synthesis of reference compounds and internal standards was based on previously reported protocols (Terzidis and Chatgilialoglu, in preparation).

### γ-radiolysis experiments

Calf thymus DNA (1 mg) was dissolved in ddH_2_O (1 mL) gently rocking overnight at 4°C. Next, the solution was transferred in a microspin filter (100 kDa) and centrifuged at ~9000 g for 2 min. To the concentrated DNA solution, 1 mL of ddH_2_O was added and the mixture was centrifuged again. The previous procedure was repeated for three times. Finally, an extra volume of ddH_2_O was added and the concentration of the final DNA solution was estimated according to its absorbance at 260 nm. In a typical gamma irradiation experiment, 200 μ L of the desalinated ctDNA solution (0.5 mg/mL) were placed in a glass vial of 2 mL, flushed with N_2_O or N_2_O/O_2_ 95:5 for 10 min. The solution was exposed to gamma rays in a ^60^Co Gammacell apparatus (dose rate 4.1 Gy/min) and the irradiation dose increased from 0 to 20, 40, and 60 Gy. In the case of the experiments with the single stranded DNA the samples were heated up to 92°C for 2 min and were cooled down rapidly in ice water prior to irradiation. All the irradiation experiments were performed in triplicates.

### Enzymatic digestion to nucleosides

**Figure 2:**
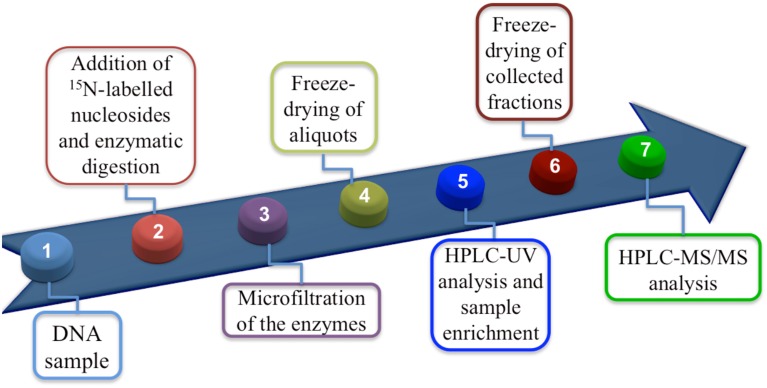
**Flow diagram showing protocol steps**.

Materials and methods are described in detail elsewhere (Terzidis and Chatgilialoglu, in preparation). Figure 2 summarizes the protocol as a flow diagram, the key step being the enzymatic digestion to obtain nucleosides in the presence of antioxidants, inert atmosphere, an efficient biocompatible metal chelator and the ^15^*N*-labeled compounds. In summary, the necessary steps for an efficient digestion are:

- in 50 μ g DNA sample
- add 100 μ L Ar flushed buffer of 10 mM Tris-HCl buffer (pH 7.9), 10 mM MgCl_2_ and 50 mM NaCl containing 0.2 mM pentostatin, 5 μ M BHT and 3 mM deferoxamine
- add the ^15^N-labeled compounds
- add a gently mixed cocktail of enzymes containing 3 U of benzonase (in 20 mM Tris HCl pH 8.0, 2 mM MgCl_2_ and 20 mM NaCl), 4 mU phosphodiesterase I, 3 U DNAse I, 2 mU of PDE II and 2 U of alkaline phosphatase.
- Incubate for 21 h at 37°C
- add 35 μ L Ar flushed buffer of 0.3 M AcONa (pH 5.6) and 10 mM ZnCl
- add a gently mixed cocktail of enzymes containing 0.5 U of Nuclease P1 (in 30 mM AcONa pH 5.3, 5 mM ZnCl_2_ and 50 mM NaCl), 4 mU phosphodiesterase II and 125 mU of DNAse II.
- Incubate for 21 h at 37°C
- add 20 μ L of 0.5 M Tris HCl pH 8.9 and incubate for 2 h at 37°C (this step is optional)
- quench the basic solution with 50 μ L of 1% formic acid (final pH ~ 7).

This procedure has been tested with purine cyclonucleosides to exclude the occurrence of any degradative process.

### LC-MS/MS analysis and quantification

#### Isotope dilution tandem mass spectrometry quantification

An LC-MS/MS system (LC, Perkin Elmer Inc., USA; triple quadrupole mass spectrometer Q-Trap 4000, AB Sciex Inc., Canada) was employed for the quantification of the lesions in the enzymatically digested DNA samples, while for the unmodified nucleosides an HPLC-UV (Agilent 1100 series, Agilent Technologies, USA) was used, as described elsewhere (Terzidis and Chatgilialoglu, in preparation). Briefly, the DNA samples, spiked with the ^15^*N*-labeled nucleosides and after being enzymatically digested, were injected in the HPLC-UV system loaded with a 4.6 mm x 150 mm Luna C18 (2) 100 Å column (5 μ min particle size, Phenomenex). During the analysis the fractions in the time windows where the lesions eluted were collected and freeze-dried. The unmodified nucleosides were quantified based on their absorption at 260 nm against their response calibration curves at the same wavelength. The collected fractions were subsequently injected to the LC-MS/MS system loaded with a 2 × 150 mm Luna C18 (2) 100 Å column (3 μ min particle size, Phenomenex) working on the multiple reaction monitoring (MRM) mode. A flow diagram showing the protocol for the quantification of the lesions is reported in Figure 2. All six lesions were found and quantified independently in all DNA samples before irradiation (control experiments). Thus, values of the control experiments were subtracted from the values recorded after the irradiation of the samples with the appropriate dose giving 0 lesions/10^6^ nucleosides (radiation induced) at 0 Gy of irradiation.

## Results

Radiolysis of neutral water leads to e^−^_aq_, HO^•^ and H^•^ as shown in eq 1. The values in parentheses represent the radiation chemical yields (*G*) in units of μ mol J^−1^. In N_2_O-saturated solution (~0.02 m of N_2_O), e^−^_aq_ are efficiently transformed into HO^•^ radical via Reaction 2 (*k*_2_ = 9.1 × 10^9^ M^−1^ s^−1^), with *G*(HO^•^) = 0.55 μmol J^−1^, i.e., HO^•^ radicals and H atoms account for 90 and 10%, respectively, of the reactive species (Buxton et al., 1988). The rate constants for the reactions of HO^•^ radicals and H atoms with DNA (Reactions 3 and 4) have been estimated to be ca. 2.5 × 10^8^ M^−1^ s^−1^ and 6 × 10^7^ M^−1^ s^−1^ per base unit, respectively (Buxton et al., 1988).

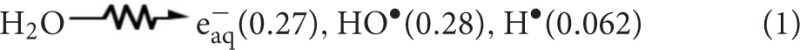



(2) $${\text{e}}_{\text{aq}}^{-}+{\text{N}}_{2}\text{O}+{\text{H}}_{2}\text{O}\to {\text{HO}}^{•}+{\text{N}}_{2}+{\text{HO}}^{-}$$

(3) $${\text{HO}}^{•}+\text{DNA}\to \text{radical product}$$

(4) $${\text{H}}^{•}+\text{DNA}\to \text{radical product}$$

**Figure 3:**
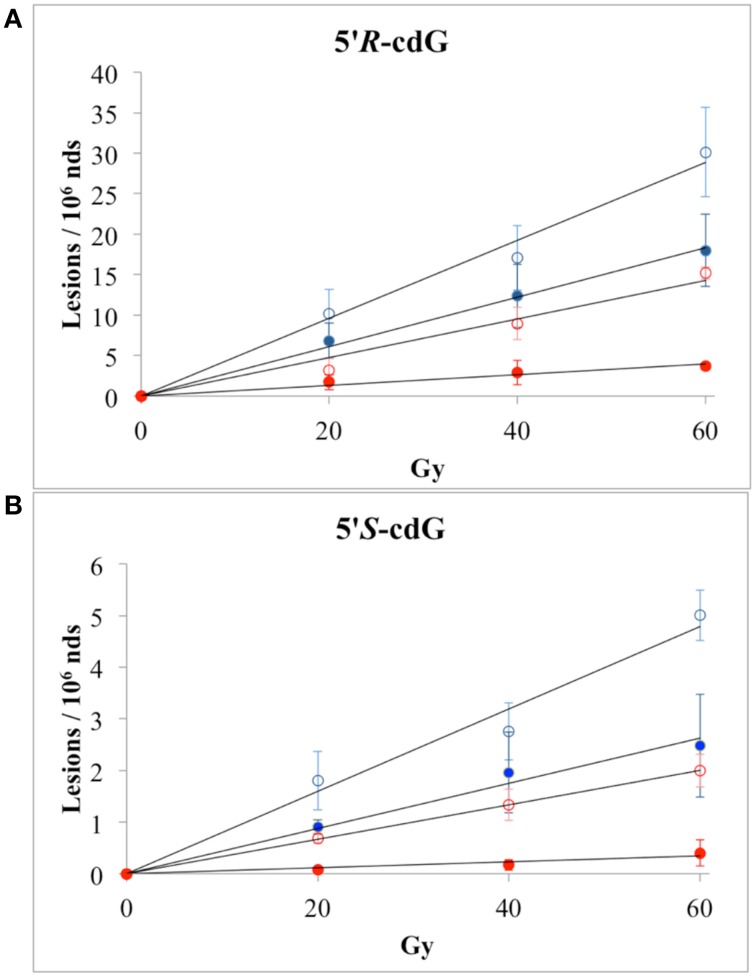
**Radiation-induced formation of 5′*R*-cdG (A) and 5′*S*-cdG (B) of ss-DNA (open circles) or ds-DNA (closed circles); (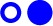) N_2_O-saturated aqueous solutions; (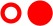) N_2_O (95%)/O_2_(5%)-saturated aqueous solutions**. The values represent the mean of *n* = 3 independent experiments.

The reactions of HO^•^ radicals with DNA were carried out using single- and double-stranded DNA. For this purpose 200 μ L of N_2_O-saturated aqueous solutions containing calf thymus DNA (0.5 mg/mL) at natural pH were irradiated under steady-state conditions with a dose rate of 4.1 Gy min^−1^, followed by enzymatic DNA digestion optimized by us and LC-MS/MS analysis (see above). Both 5′*R* and 5′*S* diastereomers of cdA and cdG were found to be generated linearly with the applied dose within the range 0–60 Gy. Figures 3A,B (blue symbols) show 5′*R-*cdG and 5′*S-*cdG, respectively, in ss-DNA (open circles) and ds-DNA (closed circles). In addition the formation of 8-oxo-7,8-dihydro-2′-deoxyguanosine (8-oxo-dG) and 8-oxo-7,8-dihydro-2′-deoxyadenosine (8-oxo-dA) was also measured. The levels of DNA lesions per Gy in the absence of oxygen are reported in Table 1 (For more details see Figures S1, S2 in Supplementary Material).

**Table 1:** **Lesions measured from irradiated sample of calf thymus DNA by LC-MS/MS**.

**Substrate^a^**	**Conditions^b^**	**Lesion**	**Lesions/10^6^Nu/Gy**	**5′R/5′S**
ss-DNA	N_2_O, 0–60 Gy	cdG	0.56	6.0
		cdA	0.48	1.7
		8-oxo-dG	49.3	
		8-oxo-dA	4.29	
	N_2_O:O_2_ (95:5), 0–60 Gy	cdG	0.24	6.5
		cdA	0.21	1.7
		8-oxo-dG	57.0	
		8-oxo-dA	3.61	
ds-DNA	N_2_O, 0–60 Gy	cdG	0.35	7.0
		cdA	0.23	1.6
		8-oxo-dG	20.2	
		8-oxo-dA	2.80	
	N_2_O:O_2_ (95:5), 0–60 Gy	cdG	0.08	7.0
		cdA	0.08	2.2
		8-oxo-dG	22.4	
		8-oxo-dA	2.37	

a Calf thymus DNA.

b Used gas and irradiation dose.

The reactions of HO^•^ radicals with ss-DNA or ds-DNA were also carried out in the presence of molecular oxygen. In particular, 200 μ L of N_2_O(95%)/O_2_(5%)-saturated aqueous solutions of calf thymus DNA (0.5 mg/mL) were irradiated under stationary state conditions with a dose rate of 4.1 Gy min^−1^. Figures 3A,B (red symbols) show 5′*R-*cdG and 5′*S-*cdG, respectively, in ss-DNA (open circles) and ds-DNA (closed circles). The levels of DNA lesions per Gy in the presence of oxygen are also reported in Table 1 (For more details see Figures S3, S4 in Supplementary Material). The 5% of oxygen-saturated solution corresponds to 6.7 × 10^−5^ M, which is the typical concentration of well-oxygenated tissues (Battino et al., 1983). Under our experimental conditions, e^−^_aq_ are still converted into HO^•^ radical via Reaction 2, although Reaction 5 is quite fast (*k*_5_ = 1.9 × 10^10^ M^−1^ s^−1^) due the large difference in concentration of the two gases and H^•^ are efficiently converted into O^−•^_2_ via Reaction 6 (*k*_6_ = 2 × 10^10^ M^−1^ s^−1^), the p*K*_*a*_(HOO^•^) being 4.8 (Buxton et al., 1988). Since O^−•^_2_ does not react with DNA, these are ideal conditions for studying the reaction of HO^•^ radicals with DNA in the presence of oxygen.

(5) $${\text{e}}_{\text{aq}}^{-}+{\text{O}}_{2}\to {\text{O}}_{2}^{-•}$$

(6) $${\text{H}}^{•}+{\text{O}}_{2}\to {\text{HOO}}^{•}⇌{\text{O}}_{2}^{-•}+{\text{H}}^{+}$$

In all cases, the formation of the 5′*R* and 5′*S* diastereomers of both cdG and cdA, as well as the 8-oxo-dG and 8-oxo-dA, was found to increase with the dose of γ-rays within the dose-range 0–60 Gy. The levels of lesions per Gy in the presence of oxygen are also reported in Table 1.

## Discussion

**Figure 4:**
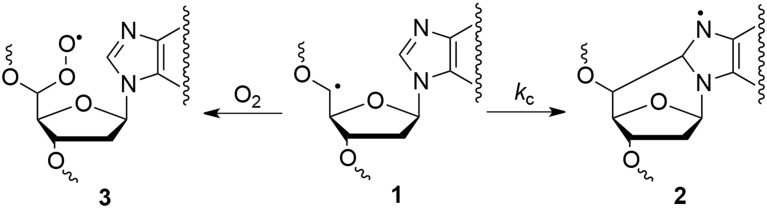
**Partition of C5′ radical 1 between cyclization reaction to give 2 and molecular oxygen addition to give 3**.

Many efforts have been devoted during the last two decades to the identification and measurement of cdA and cdG lesions in DNA samples (see Figure 1). These lesions are the products of the intramolecular attack of C5′ radical to the purine moiety. Both lesions have two diastereomeric forms (5′*R* and 5′*S*), for the configuration of the C5′ position. The order of reactivity of HO^•^ radical toward the various C–H bonds of the 2-deoxyribose moiety of DNA is expected to parallel the exposure to solvent of the 2-deoxyribose hydrogen atoms (i.e., H5′ > H4′ > H3′ ≈ H2′ ≈ H1′) (Chan et al., 2010). The yield of cdG was found to be equal or higher than that of cdA, the ratio of formation between cdG and cdA varying from 1 to 1.5 in all cases. The 5′*R* diastereomer is formed predominantly leading to 5′*R*/5′*S* ratio of ~6.5:1.0 for cdG and ~1.7:1.0 for cdA. It is worth mentioning that 5′*R*/5′*S* ratios of 8.3:1 for cdG and 6:1 for cdA were obtained in water upon irradiation of free nucleosides (Chatgilialoglu et al., 2007; Boussicault et al., 2008), indicating that the 5′*R*-diastereomer is always predominant in aqueous solution independently of the molecular complexity. On the other hand, the formation of 8-oxo-dG was about 90- and 60-fold higher than cdG in ss- and ds-DNA, respectively, in the absence of oxygen, whereas the formation of 8-oxo-dA was about 10-fold higher in both ss- and ds-DNA. The formation of both cdG and cdA decreases substantially in the presence of oxygen, indicating competition paths for C5′ radical between cyclization and addition to oxygen (Figure 4). Moreover, the 5′*R*/5′*S* ratio is similar for both cdG and cdA as observed in the absence of oxygen.

Experimental data on the reaction of HO^•^ radical with simple nucleosides like 2′-deoxyadenosine and 2′-deoxyguanosine indicated that ca. 10% of H-atom abstraction occurs at the H5′ position affording purine 5′,8-cyclonucleosides (Chatgilialoglu et al., 2007; Boussicault et al., 2008). Rate constants (*k*_*c*_) of 1.6 × 10^5^ s^−1^ and 6.9 × 10^5^ s^−1^ for the cyclization of 2′-deoxyadenosin-5′-yl and 2′-deoxyguanosin-5′-yl radicals (1→2) were also reported at room temperature (cf., Figure 4) (Chatgilialoglu et al., 2003, 2011b). The analogous cyclization rates in ss-DNA or ds-DNA are unknown. With the due precaution and reasonable assumptions, the rate constants of cyclization in ss-DNA or ds-DNA can be estimated from the data of Table 1. Assuming that in Figure 4 the radical 1 is converted quantitatively to cdA and cdG in the absence of oxygen, and that decreased formation of purine 5′,8-cyclonucleosides in the presence of oxygen is mainly due to products deriving from the peroxyl radical 3, the rate constant of unimolecular path can be estimated by applying the free-radical clock methodology (Newcomb, 1993). Using [O_2_] = 6.7 × 10^−5^ M and *k*(O_2_) = 1 × 10^9^ M^−1^ s^−1^, we estimated *k*_*c*_ to be ca. 5 × 10^4^ s^−1^ for both 2′-deoxyadenosin-5′-yl and 2′-deoxyguanosin-5′-yl radicals in ss-DNA and about halved (2–3 × 10^4^ s^−1^) in ds-DNA. We suggest that (i) these values represent the mean rate constant for cyclization obtained along the DNA sequence, and (ii) local conformations due to the supramolecular organization of DNA can give different contribution to these values, but overall they reduce substantially the cyclization rates of the two radicals.

It is worth pointing out that the data reported in the literature so far on the radiation-induced formation of purine 5′,8-cyclonucleosides in DNA give a quite confusing scenario. All data were compared in a review article (Chatgilialoglu et al., 2011a) evidencing the discrepancies including the latest one (Belmadoui et al., 2010). In Table 1, in N_2_O-saturated solution experiments the level of lesions/10^6^ nucleosides/Gy was found to be 0.23 for cdA, and 0.35 for cdG in ds-DNA, whereas the values of similar experiments were reported by one of us and others to be 14.2 and 20.1 (Belmadoui et al., 2010), which is a ~60-fold excess for both cdA and cdG. It is also worth mentioning that the 8-oxo-dA and 8-oxo-dG formation corresponds to a ~3-fold excess. These discrepancies are likely derived from the analytical steps required after the reaction, which involve the enzymatic DNA digestion and the mass spectrometry assay. Indeed, the former quantification of 5′*R* and 5′*S* diastereomers of cdA and cdG was achieved by external calibration, whereas in the present work we employed HPLC coupled with tandem mass spectrometry (LC-MS/MS) with quantification given by the isotope-dilution technique.

## Conclusions

The use of purine 5′,8-cyclonucleosides as marker of DNA damage and reporter of DNA structures at the moment of the HO^•^ insult is increasingly appreciated in free radical research, together with the advantage that cyclopurine markers do not suffer the stability problems and artifactual oxidative process of the most known 8-oxo-dG. In this paper we applied the new analytical protocol to investigate the efficacy of the HO^•^ generated by water radiolysis to form the cdA and cdG lesions in single and double stranded DNA in the presence and absence of molecular oxygen. All together our findings suggest that cdA and cdG formation depends on the dose of irradiation and the DNA secondary structure (single or double stranded), with the lesions following the order 5′*R*-cdG > 5′*R*-cdA > 5′*S*-cdA > 5′*S*-cdG. Particularly, in absence of oxygen the HO^•^ insult causes ~2 times more production of cdA and cdG in single than in double stranded DNA, with the 5′*R* diastereoisomer always more predominant than 5′*S*. Interestingly, the presence of molecular oxygen showed no influence on the cyclopurines diastereomeric ratios, but remarkably reduced their formation yields.

### Conflict of interest statement

The authors declare that the research was conducted in the absence of any commercial or financial relationships that could be construed as a potential conflict of interest.
