# Supplementary material for: Radiation-induced formation of purine lesions in single and double stranded DNA: revised quantification
Source: Front Chem. 2015 Mar 20;3:18. doi: 10.3389/fchem.2015.00018 (PMC4367438; doi:10.3389/fchem.2015.00018)
Supplement: Supplementary file 1 [file Presentation1.PDF]

Supplementary Material

Radiation-induced formation of purine lesions in single and double stranded DNA: Revised quantification

Michael A. Terzidis[1], Carla Ferreri[1], and Chryssostomos Chatgilialoglu[1,2*]

[1] Istituto per la Sintesi Organica e la Fotoreattività, Consiglio Nazionale delle Ricerche, Via P. Gobetti 101, 40129 Bologna, Italy
[2] Institute of Nanoscience and Nanotechnology, N.C.S.R. "Demokritos", 15310 Agia Paraskevi, Athens, Greece

*** Correspondence:** Chryssostomos Chatgilialoglu, Institute of Nanoscience and Nanotechnology, N.C.S.R. "Demokritos", 15310 Agia Paraskevi, Athens, Greece
c.chatgilialoglu@inn.demokritos.gr or chrys@isof.cnr.it

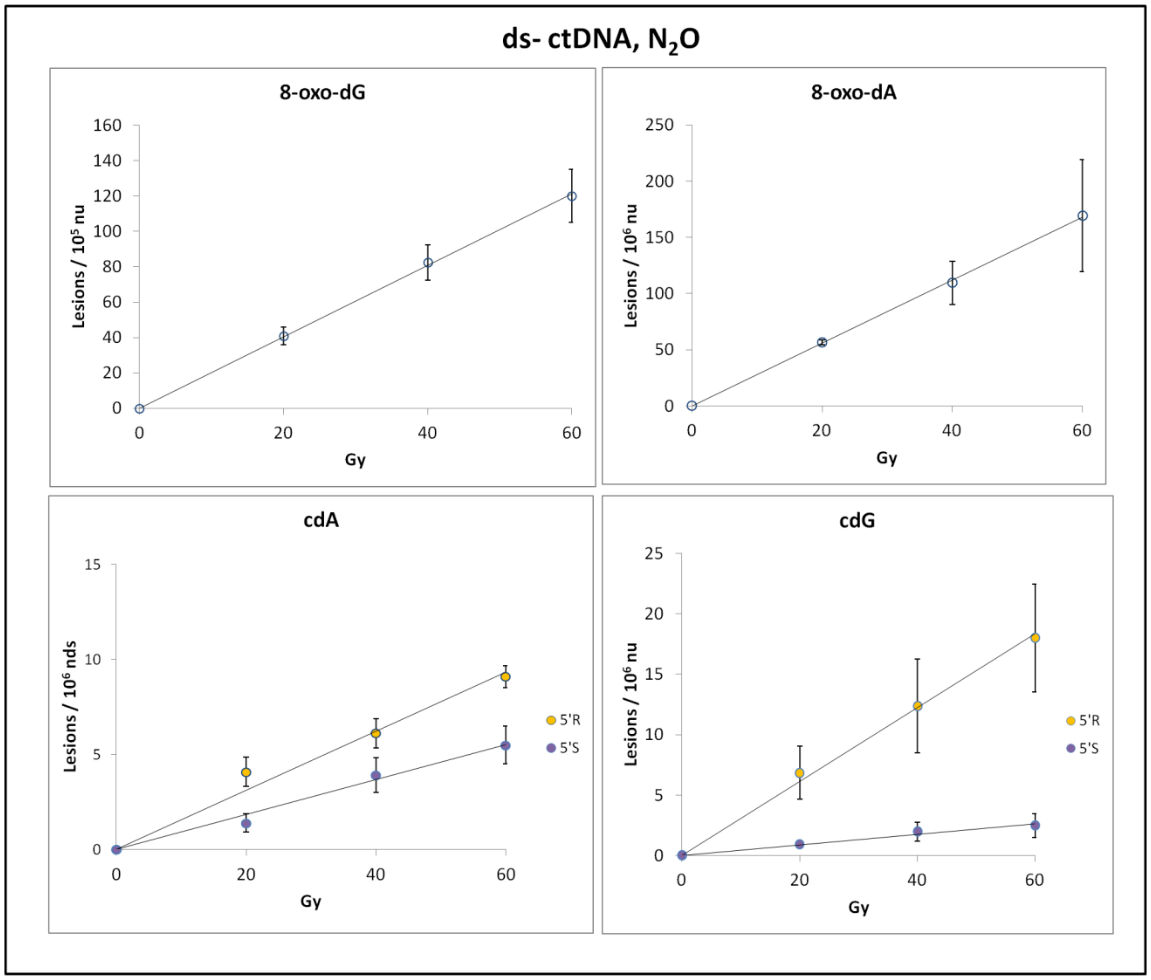

Figure S1: Radiation induced formation of 8-oxo-dG, 8-oxo-dA, (5'*R* ●, 5'*S* ●) and cdA (5'*R* ●, 5'*S* ●) in aqueous N_2O saturated of ds- ctDNA. Each data point represents the mean of *n*=3 independent experiments and the uncertainties are the standard errors. The values obtained from each independent experiment were normalized by subtracting the background levels of the lesions.

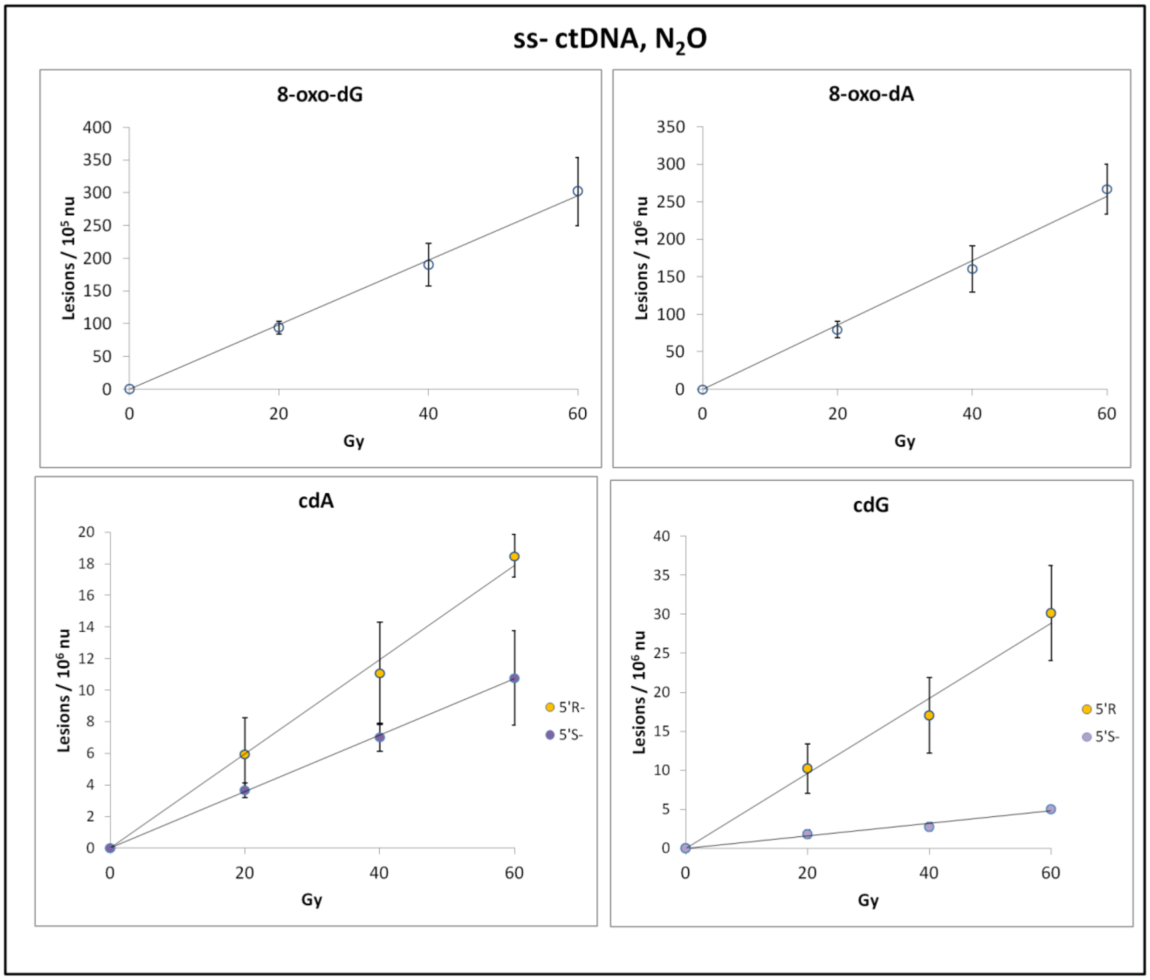

Figure S2: Radiation induced formation of 8-oxo-dG (○), 8-oxo-dA (○), cdG (5'*R* ●, 5'*S* ●) and cdA (5'*R* ●, 5'*S* ●) in aqueous N_2O saturated of ss- ctDNA. Each data point represents the mean of *n*=3 independent experiments and the uncertainties are the standard errors. The values obtained from each independent experiment were normalized by subtracting the background levels of the lesions.

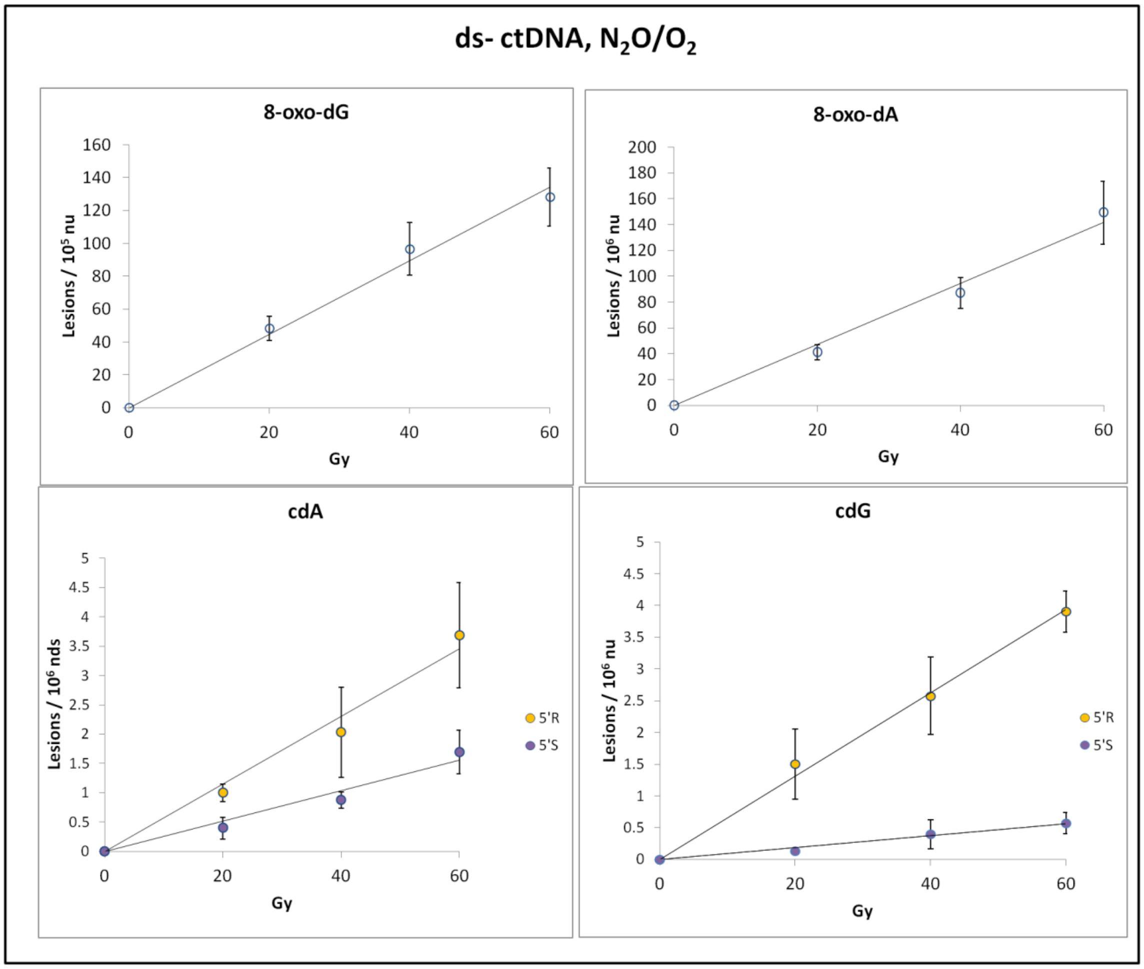

Figure S3: Radiation induced formation of 8-oxo-dG (○), 8-oxo-dA (○), cdG (5'*R* ●, 5'*S* ●) and cdA (5'*R* ●, 5'*S* ●) in aqueous N_2O(95%)/O_2(5%) saturated of ds- ctDNA. Each data point represents the mean of n=3 independent experiments and the uncertainties are the standard errors. The values obtained from each independent experiment were normalized by subtracting the background levels of the lesions.

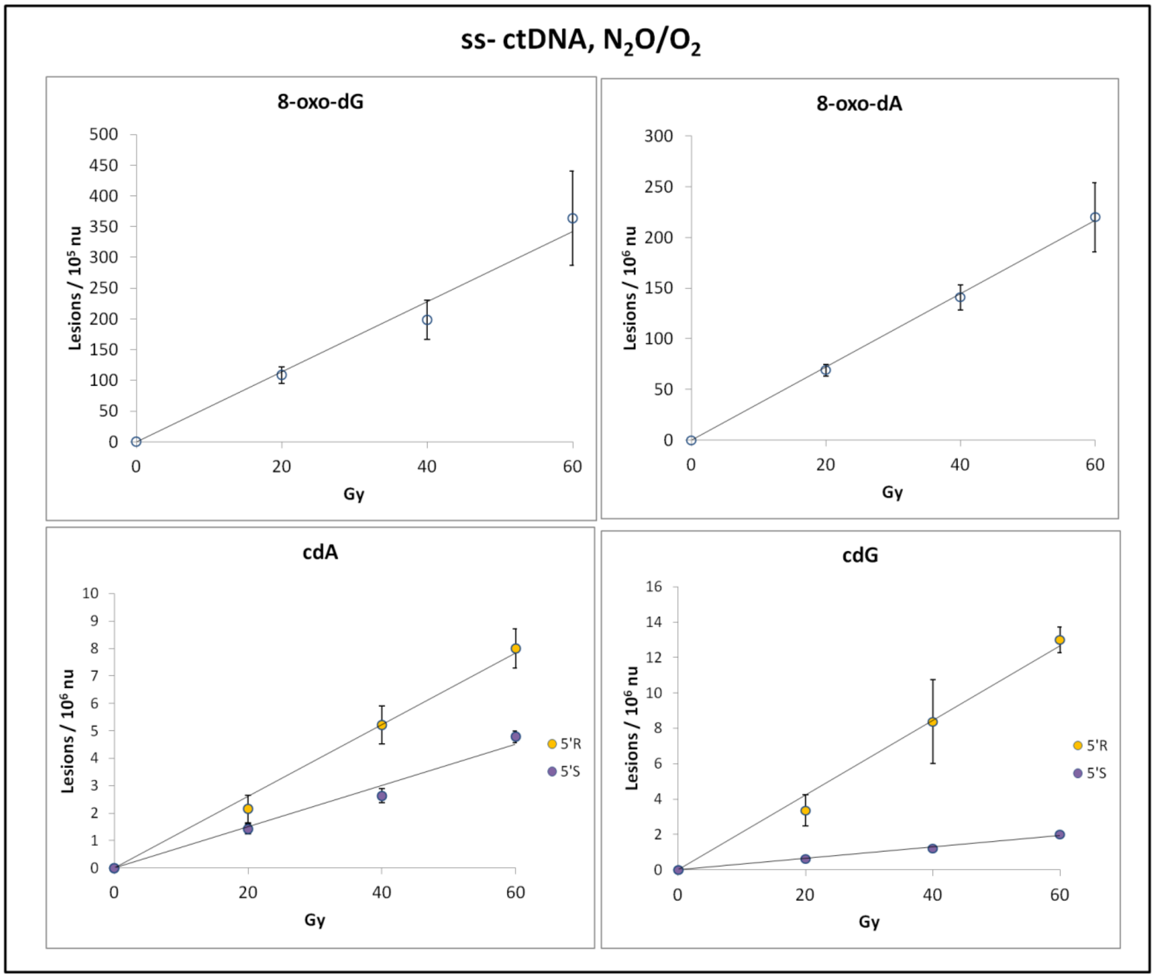

Figure S4: Radiation induced formation of 8-oxo-dG, 8-oxo-dA, cdG (5'*R* □, 5'*S* □) and cdA (5'*R* □, 5'*S* □) in aqueous N_2O(95%)/O_2(5%) saturated of ss- ctDNA. Each data point represents the mean of *n*=3 independent experiments and the uncertainties are the standard errors. The values obtained from each independent experiment were normalized by subtracting the background levels of the lesions.
